# 3-[(*R*)-3,3-Dichloro-2-hydroxy­prop­yl]-8-hydr­oxy-6-meth­oxy-1*H*-isochromen-1-one

**DOI:** 10.1107/S1600536808026391

**Published:** 2008-08-20

**Authors:** Hua-Rong Huang, Yan-Xiong Fang, Zhi-Yun Du, Kun Zhang, Yong-Cheng Lin

**Affiliations:** a Faculty of Light Industrial and Chemical Engineering, Guangdong University of Technology, Guangzhou 510090, People’s Republic of China; b School of Chemistry and Chemical Engineering, Sun Yat-Sen University, Guangzhou 510275, People’s Republic of China

## Abstract

The title compound, C_13_H_12_Cl_2_O_5_, is an isocoumarin compound which has been isolated from the ethyl acetate extract of the fermentation broth of actinomycete *Streptomyces sp*. (V_4_) from the South China Sea. There are intra- and inter­molecular hydrogen bonds and halogen bonds [Cl⋯Cl = 3.434 (2) Å; C—Cl⋯Cl = 121.6°]. The intermolecular O—H⋯O hydrogen bonds link mol­ecules into chains along the *b* axis.

## Related literature

For related literature, see: Larsen & Breinholt (1999 ▶).
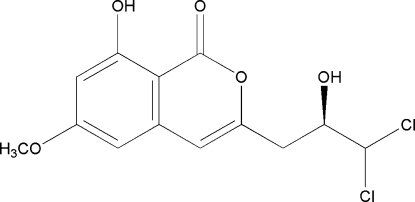


## Experimental

### 

#### Crystal data

C_13_H_12_Cl_2_O_5_
*M* *_r_* = 319.13
Monoclinic, 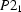

*a* = 9.483 (3) Å
*b* = 6.757 (2) Å
*c* = 10.548 (3) Å
β = 99.217 (5)°
*V* = 667.1 (4) Å^3^
*Z* = 2
Mo *K*α radiation
μ = 0.50 mm^−1^
*T* = 293 (2) K
0.50 × 0.34 × 0.21 mm

#### Data collection

Bruker SMART 1K area-detector diffractometer
Absorption correction: multi-scan (*SADABS*; Sheldrick, 1996 ▶) *T*_min_ = 0.787, *T*_max_ = 0.902
4190 measured reflections
2524 independent reflections
2320 reflections with *I* > 2σ(*I*)
*R*_int_ = 0.019

#### Refinement

*R*[*F* ^2^ > 2σ(*F* ^2^)] = 0.035
*wR*(*F* ^2^) = 0.095
*S* = 1.07
2524 reflections
184 parameters
1 restraint
H-atom parameters constrained
Δρ_max_ = 0.26 e Å^−3^
Δρ_min_ = −0.39 e Å^−3^
Absolute structure: Flack (1983 ▶), 931 Friedel pairs
Flack parameter: 0.06 (8)

### 

Data collection: *SMART* (Bruker, 1999 ▶); cell refinement: *SAINT-Plus* (Bruker, 1999 ▶); data reduction: *SAINT-Plus*; program(s) used to solve structure: *SHELXS97* (Sheldrick, 2008 ▶); program(s) used to refine structure: *SHELXL97* (Sheldrick, 2008 ▶); molecular graphics: *SHELXTL* (Sheldrick, 2008 ▶); software used to prepare material for publication: *SHELXTL*.

## Supplementary Material

Crystal structure: contains datablocks I, global. DOI: 10.1107/S1600536808026391/ww2128sup1.cif

Structure factors: contains datablocks I. DOI: 10.1107/S1600536808026391/ww2128Isup2.hkl

Additional supplementary materials:  crystallographic information; 3D view; checkCIF report

## Figures and Tables

**Table 1:** Hydrogen-bond geometry (Å, °)

*D*—H⋯*A*	*D*—H	H⋯*A*	*D*⋯*A*	*D*—H⋯*A*
O5—H5⋯O3^i^	0.82	2.18	2.913 (3)	149
O3—H3⋯O2	0.82	1.91	2.624 (2)	145

Symmetry code: (i) 
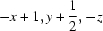
.

**Fig. 1.**
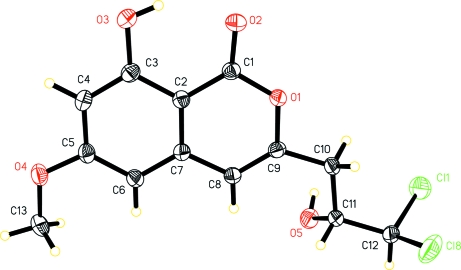
View of the molecules in the asymmetric unit of (I), with anisotropic displacement parameters drawn at the 50% probability level.

**Fig. 2.**
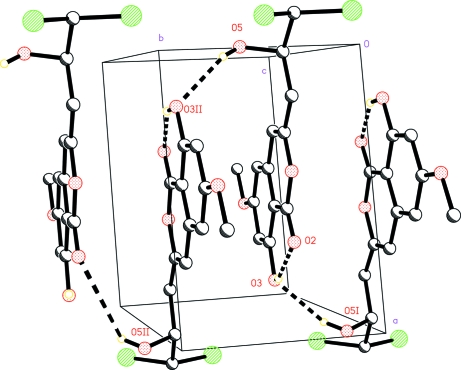
A view of the hydrogen-bonded molecular chains (Dashed lines). The chains are aligned parallel to the crystallographic b axis. H atoms not involved in H-bonding have been omitted for clarity. Symmetry codes: (I) 1 - x,y - 1/2,-z; (II) 1 - x,1/2 + y,-z.

**Fig. 3.**
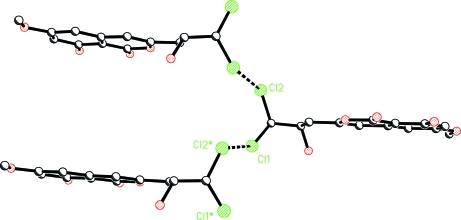
Supplementary figure.

## supplementary crystallographic information

### Comment

As a kind of natural products from marine microorganisms, 3-((*R*)-3,3-dichloro-2-hydroxypropyl)-8-hydroxy-6-methoxy-1*H*- isochromen-1-one, is a isocoumarin-related dihydrodiaportin compound, from the fermentation broth of *actinomycetes Streptomyces sp*. (V4). In the primary biotest, the title compound showed weak activity inhibiting AchE *in vitro* (IC_50_ = 3.9 uM/ml).

Larsen *et al.* also reported dichlorodiaportin (Larsen & Breinholt, 1999), but it was isolated from quite different microorganism, the typical cheese-associated isolates of *Penicillium nalgiovense*, and its structure was only elucidated based on spectral analysis, and the absolute configuration was depicted S by comparison with the optical rotation of those reported for several analogues of known absolute configuration. However, according to the optical rotation and our crystal structure, it is clear that Larsen's compound is the same compound as our dichlorodiaportin.

We report here the crystal structure of (I). Compound (I) crystallizes in space group P 2_1_, and the 8-hydroxy-isochromen-1-one core rings in (I) is a planar conjugated ring system (Fig.1). In the crystal structure, there is an intra- molecular hydrogen bond [O···O = 2.624 (2) Å, and O—H···O = 144.6°] of O—H···O type between O3 and O5 hyroxyl groups in (I), and adjacent molecules form an infinite one-dimensional chain along *b* axis *via* O—H···O intermolecular hydrogen bonds [O···O = 2.913 (3)Å, and O—H···O = 149.0°] between the O3 and O5 hydroxyl groups (Fig.1 and Table 2).

### Experimental

A strain of *streptomyces sp*. (V_4_) was isolated from the South China Sea, has been deposited in the School of Chemistry and Chemical Engineering, Sun Yat-Sen University, Guangzhou, P. R. China.

Culture conditions: soluble starch 10 g l^-1^, Ca carbonate 2 g l^-1^, ammorium sulfate 2 g l^-1^, dipotassium hydrogen phosphate 1 g l^-1^, magnesium sulfate hydrated 1 g l^-1^, sodium chloride 1 g l^-1^, yeast extract 10 g l^-1^, pH 7.2 and incubation at 298 K for 6 d. For the extraction and separation of the metabolite, the cultures (100 *L*) of (I) were filtered through cheesecloth. The filtrate was concentrated to 5*L* below 323 K, then extracted three times by shaking with an equal volume of ethyl acetate. The extract was evaporated under reduced pressure. The combined organic extracts were subjected to silica-gel column chromatography, eluting with petroleum ether/ethyl (1:1 *v*/*v*) acetate, to yield (I).

Colorless block crystals of (I) were obtained by evaporation of a methanol solution.

The compound identity was confirmed by NMR spectroscopy, Elemental Analysis, IR, FAB-MS, melting point and optical rotation.

^1^H-NMR in CDCl_3_ (500 MHz): 6.59 (s, 1H), 6,57 (d, J = 2.0 1H), 3.01 (m, 1H), 2.95 (m, 1H), 4.42 (m, 1H), 6.20 (d, J = 3.0 1H), 3.92 (s, 3H), 11.10 (s, OH) and 5.20 (d, J = 6.0, OH).

^13^C-NMR in CDCl_3_ (125 MHz): 166.8 (C), 154 (C), 107.4 (CH), 140.4 (C), 102.1 (CH), 168.0 (C), 101.2 (CH), 164.3 (C), 111.4 (C), 37.3 (CH_2_), 73.1 (CH), 77.2 (CH) and 56.3 (CH_3_).

Elemental Analysis: C 48.91, H 3.97, calc. (for C_13_H_12_O_5_C_l2_): C 48.93, H 3.79%.

IR (KBr): 3474, 3032, 2986, 1693, 1643, 1568, 1465, 1356, 1237, 1197, 1096, 853, 790, 690.

FAB-MS: 319 [*M*]^+^, 321 [*M*+2]^+^, 235 [*M*-CHCl_2_]^+^, 107, 77.

M.p. 419–420 K.

[α]^20^_D_ = +7.8 (c = 0.05, MeOH).

### Refinement

The H atoms were positioned geometrically and were treated as riding on their parent C atoms, with C—H distances in the range of 0.93–0.98 Å, with *U*_iso_ (H) = 1.2–1.5 times *U*_eq_ of the parent atom. H atoms attached to O3 and O5 (hydroxyl oxygen atoms) were located in difference Fourier maps and refined initially with distance restraints of 0.82 Å with *U*_iso_ (H) = 1.5 times *U*_eq_ (O).

### Crystal data

**Table:**

C_13_H_12_Cl_2_O_5_	*F*_000_ = 328
*M**_r_* = 319.13	*D*_x_ = 1.589 Mg m^−^^3^
Monoclinic, *P*2_1_	Melting point: 420 K
Hall symbol: P 2yb	Mo *K*α radiation λ = 0.71073 Å
*a* = 9.483 (3) Å	Cell parameters from 1002 reflections
*b* = 6.757 (2) Å	θ = 2.7–27.0º
*c* = 10.548 (3) Å	µ = 0.50 mm^−^^1^
β = 99.217 (5)º	*T* = 293 (2) K
*V* = 667.1 (4) Å^3^	Block, colorless
*Z* = 2	0.50 × 0.34 × 0.21 mm

### Data collection

**Table:**

Bruker SMART 1K area-detector diffractometer	2524 independent reflections
Radiation source: fine-focus sealed tube	2320 reflections with *I* > 2σ(*I*)
Monochromator: graphite	*R*_int_ = 0.019
*T* = 293(2) K	θ_max_ = 27.1º
φ and ω scans	θ_min_ = 2.0º
Absorption correction: multi-scan(SADABS; Sheldrick, 1996)	*h* = −12→11
*T*_min_ = 0.787, *T*_max_ = 0.902	*k* = −8→7
4190 measured reflections	*l* = −13→11

### Refinement

**Table:**

Refinement on *F*^2^	Hydrogen site location: difference Fourier map
Least-squares matrix: full	H-atom parameters constrained
*R*[*F*^2^ > 2σ(*F*^2^)] = 0.035	*w* = 1/[σ^2^(*F*_o_^2^) + (0.0525*P*)^2^ + 0.1606*P*] where *P* = (*F*_o_^2^ + 2*F*_c_^2^)/3
*wR*(*F*^2^) = 0.095	(Δ/σ)_max_ < 0.001
*S* = 1.07	Δρ_max_ = 0.26 e Å^−^^3^
2524 reflections	Δρ_min_ = −0.39 e Å^−^^3^
184 parameters	Extinction correction: none
1 restraint	Absolute structure: Flack (1983), 931 Friedel pairs
Primary atom site location: structure-invariant direct methods	Flack parameter: 0.06 (8)
Secondary atom site location: difference Fourier map	

### Special details

**Table:**

Geometry. All e.s.d.'s (except the e.s.d. in the dihedral angle between two l.s. planes) are estimated using the full covariance matrix. The cell e.s.d.'s are taken into account individually in the estimation of e.s.d.'s in distances, angles and torsion angles; correlations between e.s.d.'s in cell parameters are only used when they are defined by crystal symmetry. An approximate (isotropic) treatment of cell e.s.d.'s is used for estimating e.s.d.'s involving l.s. planes.
Refinement. Refinement of *F*^2^ against ALL reflections. The weighted *R*-factor *wR* and goodness of fit *S* are based on *F*^2^, conventional *R*-factors *R* are based on *F*, with *F* set to zero for negative *F*^2^. The threshold expression of *F*^2^ > σ(*F*^2^) is used only for calculating *R*-factors(gt) *etc*. and is not relevant to the choice of reflections for refinement. *R*-factors based on *F*^2^ are statistically about twice as large as those based on *F*, and *R*- factors based on ALL data will be even larger.

### Fractional atomic coordinates and isotropic or equivalent isotropic displacement parameters (Å^2^)

**Table:**

	*x*	*y*	*z*	*U*_iso_*/*U*_eq_	
C1	0.54937 (19)	0.4698 (4)	−0.06308 (18)	0.0334 (4)	
C2	0.55042 (19)	0.4661 (4)	0.07326 (17)	0.0317 (4)	
C3	0.6821 (2)	0.4688 (4)	0.15853 (18)	0.0356 (4)	
C4	0.6830 (2)	0.4614 (4)	0.28857 (19)	0.0387 (4)	
H4	0.7694	0.4607	0.3446	0.046*	
C5	0.5540 (2)	0.4548 (4)	0.33695 (18)	0.0361 (4)	
C6	0.4234 (2)	0.4543 (4)	0.25529 (18)	0.0369 (4)	
H6	0.3383	0.4501	0.2884	0.044*	
C7	0.42213 (19)	0.4602 (4)	0.12326 (17)	0.0332 (4)	
C8	0.29092 (19)	0.4610 (4)	0.03216 (18)	0.0358 (4)	
H8	0.2038	0.4583	0.0619	0.043*	
C9	0.29283 (18)	0.4657 (4)	−0.09298 (17)	0.0332 (4)	
C10	0.17165 (19)	0.4676 (5)	−0.20293 (17)	0.0356 (4)	
H10A	0.1783	0.3505	−0.2548	0.043*	
H10B	0.1821	0.5820	−0.2562	0.043*	
C11	0.02418 (19)	0.4735 (5)	−0.16418 (17)	0.0393 (5)	
H11	0.0161	0.3586	−0.1091	0.047*	
C12	−0.0964 (2)	0.4600 (5)	−0.2789 (2)	0.0471 (5)	
H12	−0.1876	0.4740	−0.2473	0.057*	
C13	0.4413 (3)	0.4492 (5)	0.5247 (2)	0.0502 (5)	
H13A	0.3875	0.5677	0.5015	0.075*	
H13B	0.4673	0.4429	0.6163	0.075*	
H13C	0.3843	0.3359	0.4950	0.075*	
Cl1	−0.08373 (8)	0.65243 (17)	−0.39091 (7)	0.0803 (3)	
Cl2	−0.09425 (9)	0.22660 (16)	−0.35406 (9)	0.0829 (3)	
O1	0.42041 (13)	0.4685 (3)	−0.14183 (12)	0.0349 (3)	
O2	0.65509 (15)	0.4724 (3)	−0.11589 (14)	0.0452 (4)	
O3	0.80850 (14)	0.4768 (3)	0.11445 (14)	0.0459 (4)	
H3	0.7939	0.4940	0.0365	0.069*	
O4	0.56832 (17)	0.4506 (3)	0.46640 (14)	0.0463 (4)	
O5	0.00051 (18)	0.6449 (3)	−0.09336 (15)	0.0524 (5)	
H5	0.0323	0.7420	−0.1259	0.079*	

### Atomic displacement parameters (Å^2^)

**Table:**

	*U*^11^	*U*^22^	*U*^33^	*U*^12^	*U*^13^	*U*^23^
C1	0.0317 (8)	0.0335 (10)	0.0355 (9)	0.0005 (10)	0.0067 (7)	−0.0017 (10)
C2	0.0315 (8)	0.0309 (9)	0.0325 (9)	0.0027 (10)	0.0044 (7)	0.0009 (9)
C3	0.0321 (9)	0.0324 (10)	0.0414 (10)	0.0010 (11)	0.0034 (7)	−0.0001 (10)
C4	0.0363 (9)	0.0401 (10)	0.0367 (10)	0.0003 (11)	−0.0033 (7)	−0.0017 (11)
C5	0.0442 (10)	0.0323 (10)	0.0305 (9)	−0.0006 (11)	0.0016 (7)	−0.0008 (10)
C6	0.0364 (9)	0.0434 (11)	0.0316 (9)	0.0011 (11)	0.0072 (7)	0.0010 (10)
C7	0.0323 (9)	0.0363 (10)	0.0309 (9)	−0.0008 (11)	0.0046 (7)	−0.0008 (10)
C8	0.0287 (8)	0.0461 (11)	0.0330 (9)	0.0007 (11)	0.0067 (7)	0.0016 (11)
C9	0.0297 (8)	0.0352 (10)	0.0354 (9)	−0.0024 (10)	0.0070 (7)	−0.0031 (10)
C10	0.0330 (8)	0.0470 (11)	0.0271 (8)	0.0013 (11)	0.0058 (6)	−0.0018 (10)
C11	0.0333 (9)	0.0568 (13)	0.0281 (9)	−0.0076 (11)	0.0060 (7)	−0.0016 (11)
C12	0.0311 (9)	0.0760 (16)	0.0349 (10)	−0.0087 (14)	0.0074 (7)	−0.0079 (13)
C13	0.0613 (13)	0.0601 (15)	0.0300 (10)	0.0009 (15)	0.0095 (9)	0.0023 (11)
Cl1	0.0525 (4)	0.1251 (9)	0.0574 (4)	−0.0046 (4)	−0.0090 (3)	0.0333 (5)
Cl2	0.0561 (4)	0.1063 (7)	0.0842 (5)	−0.0205 (4)	0.0046 (4)	−0.0486 (5)
O1	0.0296 (6)	0.0467 (8)	0.0291 (6)	0.0006 (8)	0.0062 (5)	−0.0002 (7)
O2	0.0332 (7)	0.0632 (10)	0.0413 (7)	0.0014 (9)	0.0126 (6)	0.0000 (9)
O3	0.0313 (6)	0.0623 (10)	0.0434 (8)	0.0011 (9)	0.0038 (5)	0.0036 (9)
O4	0.0511 (8)	0.0562 (9)	0.0298 (7)	0.0021 (10)	0.0011 (6)	0.0012 (8)
O5	0.0412 (8)	0.0737 (13)	0.0441 (8)	0.0008 (9)	0.0120 (6)	−0.0175 (9)

### Geometric parameters (Å, °)

**Table:**

C1—O2	1.223 (2)	C9—C10	1.496 (2)
C1—O1	1.364 (2)	C10—C11	1.519 (2)
C1—C2	1.437 (3)	C10—H10A	0.9700
C2—C7	1.402 (2)	C10—H10B	0.9700
C2—C3	1.417 (2)	C11—O5	1.415 (3)
C3—O3	1.354 (2)	C11—C12	1.529 (3)
C3—C4	1.371 (3)	C11—H11	0.9800
C4—C5	1.400 (3)	C12—Cl2	1.766 (3)
C4—H4	0.9300	C12—Cl1	1.774 (3)
C5—O4	1.351 (2)	C12—H12	0.9800
C5—C6	1.390 (3)	C13—O4	1.437 (3)
C6—C7	1.391 (3)	C13—H13A	0.9600
C6—H6	0.9300	C13—H13B	0.9600
C7—C8	1.445 (2)	C13—H13C	0.9600
C8—C9	1.323 (3)	O3—H3	0.8200
C8—H8	0.9300	O5—H5	0.8200
C9—O1	1.389 (2)		
			
O2—C1—O1	116.32 (17)	C9—C10—H10A	108.6
O2—C1—C2	125.56 (17)	C11—C10—H10A	108.6
O1—C1—C2	118.12 (16)	C9—C10—H10B	108.6
C7—C2—C3	119.41 (16)	C11—C10—H10B	108.6
C7—C2—C1	120.64 (16)	H10A—C10—H10B	107.6
C3—C2—C1	119.94 (16)	O5—C11—C10	113.2 (2)
O3—C3—C4	118.72 (17)	O5—C11—C12	107.8 (2)
O3—C3—C2	121.39 (17)	C10—C11—C12	112.93 (15)
C4—C3—C2	119.89 (17)	O5—C11—H11	107.6
C3—C4—C5	119.98 (17)	C10—C11—H11	107.6
C3—C4—H4	120.0	C12—C11—H11	107.6
C5—C4—H4	120.0	C11—C12—Cl2	110.3 (2)
O4—C5—C6	124.15 (19)	C11—C12—Cl1	111.17 (19)
O4—C5—C4	114.65 (17)	Cl2—C12—Cl1	110.42 (12)
C6—C5—C4	121.19 (17)	C11—C12—H12	108.3
C5—C6—C7	118.97 (18)	Cl2—C12—H12	108.3
C5—C6—H6	120.5	Cl1—C12—H12	108.3
C7—C6—H6	120.5	O4—C13—H13A	109.5
C6—C7—C2	120.54 (17)	O4—C13—H13B	109.5
C6—C7—C8	122.30 (17)	H13A—C13—H13B	109.5
C2—C7—C8	117.15 (16)	O4—C13—H13C	109.5
C9—C8—C7	121.02 (17)	H13A—C13—H13C	109.5
C9—C8—H8	119.5	H13B—C13—H13C	109.5
C7—C8—H8	119.5	C1—O1—C9	121.57 (14)
C8—C9—O1	121.48 (16)	C3—O3—H3	109.5
C8—C9—C10	129.93 (17)	C5—O4—C13	118.50 (15)
O1—C9—C10	108.59 (15)	C11—O5—H5	109.5
C9—C10—C11	114.68 (15)		
			
O2—C1—C2—C7	−179.0 (3)	C1—C2—C7—C8	−0.9 (4)
O1—C1—C2—C7	0.4 (4)	C6—C7—C8—C9	−179.7 (3)
O2—C1—C2—C3	1.1 (4)	C2—C7—C8—C9	0.5 (4)
O1—C1—C2—C3	−179.5 (2)	C7—C8—C9—O1	0.4 (4)
C7—C2—C3—O3	−179.2 (2)	C7—C8—C9—C10	−179.9 (3)
C1—C2—C3—O3	0.7 (4)	C8—C9—C10—C11	2.8 (5)
C7—C2—C3—C4	1.4 (4)	O1—C9—C10—C11	−177.6 (2)
C1—C2—C3—C4	−178.7 (2)	C9—C10—C11—O5	61.4 (3)
O3—C3—C4—C5	179.4 (2)	C9—C10—C11—C12	−175.8 (3)
C2—C3—C4—C5	−1.2 (4)	O5—C11—C12—Cl2	−168.62 (15)
C3—C4—C5—O4	−179.1 (3)	C10—C11—C12—Cl2	65.6 (3)
C3—C4—C5—C6	0.4 (4)	O5—C11—C12—Cl1	68.5 (2)
O4—C5—C6—C7	179.6 (2)	C10—C11—C12—Cl1	−57.3 (3)
C4—C5—C6—C7	0.1 (4)	O2—C1—O1—C9	−180.0 (2)
C5—C6—C7—C2	0.1 (4)	C2—C1—O1—C9	0.6 (4)
C5—C6—C7—C8	−179.7 (3)	C8—C9—O1—C1	−1.0 (4)
C3—C2—C7—C6	−0.8 (4)	C10—C9—O1—C1	179.3 (2)
C1—C2—C7—C6	179.3 (3)	C6—C5—O4—C13	−1.2 (4)
C3—C2—C7—C8	179.0 (2)	C4—C5—O4—C13	178.3 (3)

### Hydrogen-bond geometry (Å, °)

**Table:**

*D*—H···*A*	*D*—H	H···*A*	*D*···*A*	*D*—H···*A*
O5—H5···O3^i^	0.82	2.18	2.913 (3)	149
O3—H3···O2	0.82	1.91	2.624 (2)	145

Symmetry codes: (i) −*x*+1, *y*+1/2, −*z*.
